# Modified technique for transscleral fixation of posterior chamber intraocular lenses

**DOI:** 10.1186/s12886-015-0118-8

**Published:** 2015-10-02

**Authors:** Chongde Long, Yantao Wei, Zhaohui Yuan, Zhiqing Zhang, Xiaofeng Lin, Bingqian Liu

**Affiliations:** From the State Key Laboratory of Ophthalmology, Zhongshan Ophthalmic Center, Sun Yat-sen University, 54 South Xianlie Road, Guangzhou, 510060 Guangdong China

**Keywords:** Aphakic eye, Posterior chamber intraocular lens, Transsclera suture fixation, Suture exposure

## Abstract

**Background:**

Suture exposure remains to be a potential problem of transscleral fixated posterior chamber intraocular lens (PCIOL). We report a modified technique to minimize the risk of suture exposure for the transscleral fixation of PCIOL.

**Methods:**

The modified surgical technique is as following: at first, two 3 mm × 4 mm square scleral pockets were created from groove incisions at opposite positions. A straight needle attached to a 10–0 polypropylene suture was passed through one incision groove. Then, a 27-Gauge hollow needle passed through the opposite sclera incision bed was used to retrieve the straight fine needle via its barrel. The sutures were tied to themselves after one more bite on the scleral bed. At last, the suture ends were left long (about 4 mm) and laid flat into corresponding laminar scleral pockets. This modified technique of PCIOL was performed in 48 post-traumatic aphakic vitrectomized eyes from 48 patients (47 male, one female) with mean age of 34.8 ± 14.8 years. Main outcome measures included best corrective visual acuity (BCVA), IOL decentration, IOL tilt, and postoperative complications.

**Results:**

The mean follow-up was 32.3 ± 10.8 months (3–67 months). The LogMAR BCVA remained stable, from a preoperative value of 0.46 ± 0.34 to postoperative 0.44 ± 0.34 (*p* = 0.69). Mild IOL tilt (5–10°) was observed in five eyes, and slight IOL decentration (0.5–1.0 mm) was seen in three cases. No case of suture exposure, suture breakage, IOL dislocation, or endophthalmitis was observed during the follow up period.

**Conclusion:**

The modified technique allowed stable placement of PCIOLs in post-traumatic aphakic eyes with a wide range of follow-up. Our procedure might have the potential benefit to avoid suture exposure in scleral-fixated IOL implantation.

**Electronic supplementary material:**

The online version of this article (doi:10.1186/s12886-015-0118-8) contains supplementary material, which is available to authorized users.

## Background

There are several surgical techniques for intraocular lens (IOL) implantation in the absence of capsular support [1]. Transsclerally fixated posterior chamber intraocular lens (PCIOL) is an effective way to correct aphakia [2–11], especially for those patients with corneal disease, iris tissue damage, angle abnormalities, or glaucoma. Being located in a position closest to the original lens, PCIOL possesses several inherent advantages: it does not contact with corneal endothelium or trabecular meshwork; acts as a mechanical barrier between vitreous cavity and anterior chamber. However, tie erosion and suture exposure remains to be a potential problem in transsclerally sutured PCIOL [12, 13]. The reported incidence rate of suture exposure was from 6.7 to 73.0 % [12–18].

Several techniques have been described to avoid suture erosion as there is a potential risk to develop endophthalmitis and IOL dislocation, including rotation the suture knots into the eye [8, 19], suturing within a scleral groove [20], burying the suture ends into scleral tunnel [21], covering the suture ends with fascia lata [6], tenon’s capsule [22], scleral flap [10, 23–27] or scleral pocket [28]. The aim of this study was to assess the long-term safety and efficacy of a variation of the sclera-covering technique for suture fixation of PCIOLs in a series of post-traumatic aphakic eyes, in which the suture ends were left long and laid flat in laminar scleral pockets, minimizing the risk for suture exposure.

## Methods

This study was conducted in compliance with the principles of the Declaration of Helsinki and was approved by the Ethics Committee of Zhongshan Ophthalmic Center, Sun Yat-sen University. Informed written consent was obtained from all the patients or guardians prior to surgery.

The inclusion criteria: total lens capsule absence in post-traumatic eyes; the minimal time between the primary surgery (pars plana vitrectomy or wound closure) and the PCIOL implantation was 3 months; the retina of the aphakic eye remained attached; no active inflammation was found in the aphakic eyes. A retrospective chart review was conducted of transscleral sutured PCIOL implantation in post-traumatic aphakic eyes performed by single surgeon (C.L.) from May 2007 to December 2013 using the new technique, which was designed to minimize the risk of suture exposure. The capsule, including the posterior and the anterior capsule, was totally compromised in all of the cases. Data collected included, ocular trauma history, preoperative and final BCVA, preoperative and postoperative intraocular pressure (IOP), and postoperative complications.

## Surgical technique

**Fig. 1:**
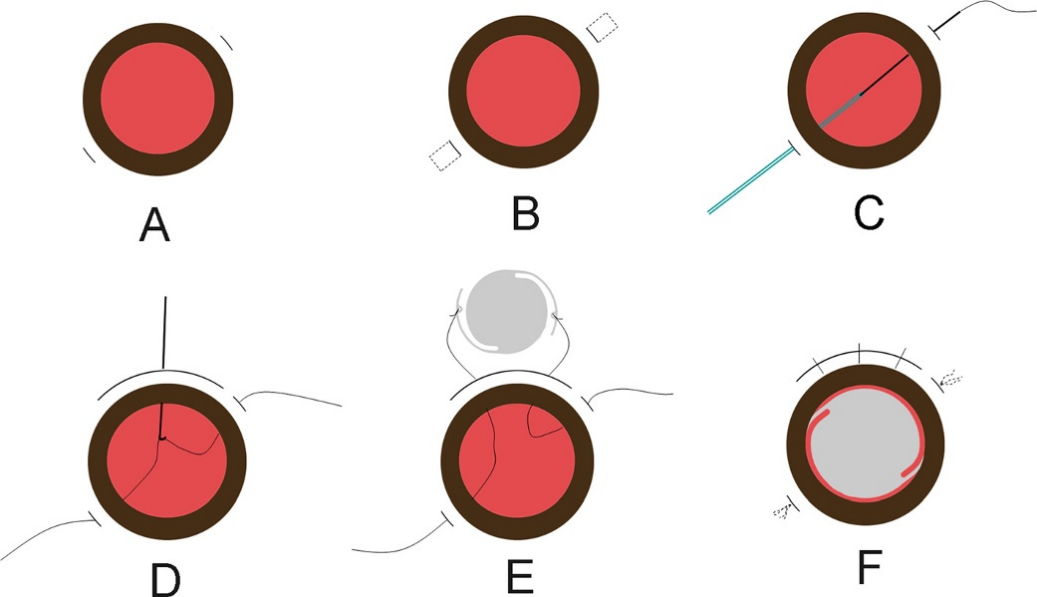
Schematic procedures of modified technique for PCIOL fixation. **a** Two 3 mm limbus-parallel half-thickness scleral incisions were made directly opposite each other, at 2 o’clock and 8 o’clock, 1.5 mm behind the limbus. **b** Two 3 mm × 4 mm scleral pockets were created with a blade to extend the incision bed posteriorly. **c** A straight needle attached to a 10–0 polypropylene suture was passed through the bed of laminar scleral incision at 2 o’clock. A 27-Gauge hollow needle passed through the opposite sclera incision bed at 8 o’clock was used to retrieve the straight fine needle via its barrel. **d** A superior corneoscleral incision was made. The suture loop was retrieved through the superior limbal wound. **e** The loop was cut and the ends were tied to the haptics of the IOL respectively. **f** A PCIOL was inserted into posterior chamber through the superior incision, and placed in the ciliary sulcus followed by pulling the sutures to center the optics. The superior corneoscleral wound was closed. The sutures were tied to themselves after one more bite on the scleral bed. The suture ends were left long (4 mm) and laid flat into the scleral pockets

**Fig. 2:**
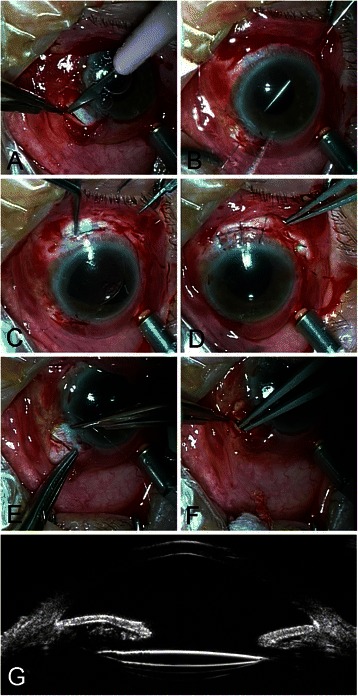
Modified technique for PCIOL fixation. **a** A scleral pocket was created with a blade. **b** A hollow needle passed through the opposite sclera incision bed to retrieve the straight fine needle via its barrel. **c** A PCIOL was inserted into posterior chamber through the superior incision. **d** The superior corneoscleral wound was closed. **e** One tick bite on the scleral bed was done with the short needle connected to the suture. **f** The suture ends were laid flat into the scleral pocket. **g** A typical UBM imaging with a PCIOL implanted

All the surgeries were performed by the same surgeon (C.L.). The surgical technique was individualized because of the trauma history and the complicated nature of the eyes resulted from previous surgery. For eyes with a history of pars plana vitrectomy 42/48 (87.5 %), a 23- or 20-Gaugue infusion cannula was inserted to maintain the intraocular pressure at 3.5 mm posterior to the limbus into the eye to prevent intraoperative globe collapse. The surgical steps (Additional file 1) are shown below:

Two 3 mm limbus-parallel half-thickness scleral incisions (1.5 mm behind the posterior surgical limbus) were made directly opposite each other (Fig. 1a). Then, two 3 mm × 4 mm scleral pockets were created with a blade to extend the incision bed posteriorly (Figs. 1b and 2a).

A straight needle attached to a 10–0 polypropylene suture was passed through the bed of one laminar scleral incision (1 o’clock). The needle was placed perpendicular to the sclera wall and then parallel to the iris until its tip appeared in the center of the pupil. A 27-Gauge hollow needle passed through the opposite sclera incision bed (1.5 mm behind the posterior surgical limbus at 7 o’clock) was used to retrieve the straight fine needle via its barrel (Figs. 1c and 2b). The straight fine needle was withdrawn from the eye guided by the hollow needle, leaving the 10–0 suture traversing the eye from one scleral bed to the other.

A superior corneoscleral incision was fashioned for implantation of the IOL. The suture loop was retrieved through the superior limbal wound (Fig. 1d). The loop was cut and the ends were tied to the haptics of the IOL respectively (Fig. 1e). The IOL was inserted into posterior chamber through the superior incision (Fig. 2c), and placed in the ciliary sulcus followed by pulling the sutures to center the optics. Severe iris and pupil damage was repaired. The superior corneoscleral wound was closed (Fig. 2d). The sutures were pulled gently and tied to themselves after one more bite on the scleral bed (Fig. 2e). The bite was placed 1.5–2 mm behind the posterior surgical limbus. The knot was tied right in the incision level or slightly behind the incision groove. The suture ends were left long (4 mm) and laid flat into the prepared scleral pockets (Figs. 1f and 2f).

The infusion cannula was removed, the conjunctival peritomy was closed. A subconjunctival injection with 20,000 unit tobramycin and 2.5 mg dexamethasone was performed at the end of the procedure. Postoperatively, topical antibiotic/steroid drops were given four times a day, antibiotic/steroid ointment was instilled at night, for 4 weeks.

## Results

The information of included eyes is presented in Table 1. This technique was successfully used in 48 patients (47 male, one female; mean age ± SD, 34.8 ± 14.8 years; range, 8–60 years) presenting with absence of lens capsule due to trauma (open globe injury 43/48, closed globe injury 5/48) between May 2007 and December 2013. Cornea scar was presented in 35/48 (72.92 %) patients. The mean interval from the primary surgery, pars plana vitrectomy or wound closure, to the secondary IOL implantation was 4.5 ± 1.9 months. Three kinds of PCIOL were used in our cohort, including PMMA single-piece posterior chamber lenses (CZ70BD, Alcon) in 35/48 eyes, foldable acrylic posterior chamber lenses (AR40e, Advanced Medical Optics) in 8/48 eyes, and black diaphragm aniridia intraocular lens (Type 67G, Morcher) in 5/48 eyes. The mean follow-up was 32.3 ± 10.8 months (range 3–67 months), 37/48 (77.08 %) patients have a follow-up period over 12 months.

**Table 1:** Patient information, types of implanted PCIOLs, follow-up period and best corrected visual acuity

Gender	
Male	47/48
Female	1/48
Mean Age	34.8 ± 14.8 (range 8–60) years
Primary trauma type	
Open globe injury	43/48 (89.6 %)
Closed globe injury	5/48 (10.4 %)
History of pars plana vitrectomy	42/48 (87.5 %)
Mean Interval from primary to PCIOL surgery	4.5 ± 1.9 months
Type of implanted IOL	
CZ70BD	35/48 (72.9 %)
AR40e	8/48 (16.7 %)
Type 67G	5/48 (10.4 %)
Mean follow-up	32.3 ± 10.8 (range 3–67) months
Follow-up >12 months	37/48 (77.1 %)
Follow-up <12 months	11/48 (22.9 %)
Best corrected Visual acuity (LogMAR)	(*p* = 0.69)
Pre-operation	0.46 ± 0.34
Post-operation	0.44 ± 0.34

Postoperative complications are shown in Table 2. Postoperatively, transient corneal edema in 37/48 eyes (77.1 %), temporary hypotony (6–10 mmHg) in 11/48 eyes (22.9 %), vitreous hemorrhage in 4/48 cases (8.3 %), temporary intraocular pressure elevation in 8/48 eyes (16.7 %), and cystoid macular edema in 5/48 cases (10.4 %) were observed. All these complications were resolved within 4 weeks. Suprachoroidal hemorrhage was observed in 2/48 eyes (4.2 %). In the follow-up period, retinal detachment was found in 2/48 eyes (4.2 %), which were reattached by pars plana vitrectomy and silicon oil tamponade. The mean best corrected visual acuity (LogMAR) remained stable, from 0.46 ± 0.34 preoperatively to 0.44 ± 0.34 postoperatively (*T*-test, *p* = 0.69). Mild IOL tilt (5–10°) was observed in five eyes, and slight IOL decentration (0.5–1.0 mm) was seen in three cases, detected by Ultrasound Biomicroscope. Figure 2g shows a typical post-operative UBM imaging, the IOL was well centered without tilt. In our case series, there were no cases of suture exposure, suture breakage, IOL dislocation, or endophthalmitis during the follow-up period.

**Table 2:** Post-operation complications

Post-operation complications	N. (%)
Transient corneal edema	37/48 (77.1 %)
Temporary hypotony	11/48 (22.9 %)
Vitreous hemorrhage	4/48 (8.3 %)
Temporary intraocular pressure elevation	8/48 (16.7 %)
Cystoid macular edema	5/48 (10.4 %)
Suprachoroidal hemorrhage	2/48 (4.2 %)
Retinal re-detachment	2/48 (4.2 %)
IOL tilt (5–10°)	5/48 (10.4 %)
IOL decentration (0.5–1.0 mm)	3/48 (6.3 %)

## Discussion

Numerous techniques of transscleral fixation of PCIOL have been developed [1]. Covering the suture end with a triangular scleral flap is one of the most common techniques. However, sclera flaps for knot coverage tends to atrophy with time [1, 12–18], probably because the area of the flap is too small. Our technique allowed long-term stable placement of PCIOLs, the roof of the scleral pocket offers a greater surface area to cover the suture ends compared with traditional triangular scleral flap. Hoffman et al. [28] described a similar technique in 2006, in which the sclera pockets were initiated from peripheral clear corneal incisions. However, their technique required two suture passes through the sclera for each haptic, creating twice as many the potential events of vitreous hemorrhage compared with single suturing technique. Our modified procedure facilitated the haptic fixation with a single transscleral pass, and also eliminated the need for suture closure of scleral flap.

An appropriate length of suture end is important to maintain the knot integrity. In theory, a longer suture end is less likely to untie spontaneously, and easier to be laid flat, thus has less chance to break or penetrate the covering-sclera. Using a longer suture end to avoid erosion in PCIOL fixation had been described by Smiddy et al. [4] in 1990 and Chen et al. [27] in 2007. With the length being about 4 mm, the suture end could be covered totally by the scleral pocket, and we found the suture end did not necessarily keep straight under the sclera roof. With this technique, no case of suture breakage was observed in our series during the follow up period.

In the most cases, we used IOLs (Alcon CZ70BD and Type 67G) with a large optic, longer haptics and two eyelets for passing suture thread. They are the appropriate IOLs specially designed for transscleral fixation. But limitation also remains in such kind of rigid IOL: it needs a much bigger incision for the IOL to enter anterior chamber. It is known that bigger incision is associated with more chances of low intraocular pressure, choroidal hemorrhage, and post-operative corneal astigmatism. AR40e is not specially designed for transscleral fixation, but it has been reported to be fixated successfully through sclera [29]. We used foldable IOL (AR40e) for the following reasons: it could be implanted through a small incision, which is associated with better intraocular pressure control, and less chance of choroidal hemorrhage; its haptics are long enough to falicitate suture fixation; the end of haptics could be enlarged through cauterizing slightly to avoid suture slippage.

Limitations of this report include: it is a retrospective study; there was a wide range of the follow-up period in the include patients; possible disadvantage of our technique include sclera pocket associated hemorrhage during operation, and potential complications correlated with intraocular suture ends at haptics fixation sites; one other drawback, as compared to Hoffman pockets, a conjunctival peritomy was needed in our technique. Hoffman’s pockets are likely to be better in preserving conjunctiva, which may increase postoperative comfort and be helpful if glaucoma surgery is needed at a later date.

Recently, sutureless technique for PCIOLs implantation [30–36] is becoming popular. Agarwal et al. achieved sutureless by using fibrin glue to close the scleral flaps [36] without suture-related complications. However, fibrin glue might be not available everywhere. Wilgucki et al. [37] used a 20 gauge blade to create ciliary sulcus-based sclerotomies to facilitate haptics passing through. The potential events of vitreous hemorrhage may be more than our procedure as the incision number and width are bigger than ours. Three of the 12 cases showed IOL dislocation one year after surgery. Ohta et al. [35] reported a Y-fixation technique without using fibrin sealant. But the flap and the groove were closed with nonabsorbable suture to prevent slippage, which was suspected to be a modified suture technique. We prefer suture fixation technique in traumatic aphakic eyes because: none of the commercially available IOLs at present is specially designed for sutureless fixation; sutureless technique requires more transscleral penetrations, which might be associated with more chances of intra-operative and post-operative hypotony, vitreous hemorrhage, and post-operative inflammation; most of the afflicted patients were work population or at a younger age, less transscleral penetration might be associated with reduced suture-related inflammation; suture fixation is more secure to withstand potential reoperation procedures including retinal retachment, sclera compression, and silicone oil tamponade in post-traumatic aphakic eyes. Modification of the present foldable IOLs might facilitate the suture fixation procedure in aphakic eyes. Further prospective clinical trials might provide more evidences for clinical surgeons by comparing the rigid hard IOLs and foldable IOLs, and comparing our technique against the sutureless technique on IOL decentration, IOL tilt, and postoperative complications.

## Conclusion

The modified technique allowed stable placement of PCIOLs in series of post-traumatic aphakic eyes with a wide range of follow-up period. According to our data, covering the suture ends with scleral pockets is an alternative way to avoid suture exposure in scleral-fixated IOL implantation. Our approach could be modified and used for other kinds of IOL or intraocular devices that requires transscleral suture fixation.

## Additional file

Additional file 1: **Video illustration of the modified technique for PCIOL suture fixation.** (MP4 16086 kb)

## Abbreviations

PCIOL: Posterior chamber intraocular lens
IOL: Intraocular lens
BCVA: Best corrective visual acuity
logMAR: Logarithm of the minimum angle of resolution
